# Nonribosomal antibacterial peptides isolated from *Streptomyces agglomeratus* 5-1-3 in the Qinghai-Tibet Plateau

**DOI:** 10.1186/s12934-023-02018-0

**Published:** 2023-01-06

**Authors:** Kan Jiang, Ximing Chen, Wei Zhang, Yehong Guo, Guangxiu Liu

**Affiliations:** 1 College of Agronomy, State Key Laboratory of Aridland Crop Science, Gansu Agricultural University, Lanzhou, 730070 Gansu China; 2 Key Laboratory of Desert and Desertification, Northwest Institute of Eco-Environment and Resources, Chinese Academy of Sciences, Lanzhou, 730030 Gansu China; 3 Key Laboratory of Extreme Environmental Microbial Resources and Engineering, Lanzhou, 730030 Gansu China

**Keywords:** *S. agglomeratus* 5-1-3, Antibacterial, Echinomycin, Nonribosomal peptides, MRSA

## Abstract

**Background:**

New antibiotics are urgently needed in clinical treatment of superdrug-resistant bacteria. Nonribosomal peptides (NRPs) are a major source of antibiotics because they exhibit structural diversity, and unique antibacterial mechanisms and resistance. Analysis of gene clusters of *S. agglomeratus* 5-1-3 showed that Clusters 3, 6, 12, 21, and 28 were used to synthesize NRPs. Here, we examined secondary metabolites of *S. agglomeratus* 5-1-3 isolated from soils in the Qinghai-Tibet Plateau, China, for NRPs with antibacterial activity.

**Results:**

We isolated a total of 36 *Streptomyces* strains with distinct colony morphological characteristics from 7 soil samples. We screened 8 *Streptomyces* strains resistant to methicillin-resistant *Staphylococcus aureus* (MRSA). We then selected *S. agglomeratus* 5-1-3 for further study based on results of an antibacterial activity test. Here, we isolated three compounds from *S. agglomeratus* 5-1-3 and characterized their properties. The crude extract was extracted with ethyl acetate and purified with column chromatography and semipreparative high-performance liquid chromatography (HPLC). We characterized the three compounds using NMR analyses as echinomycin (**1**), 5,7,4ʹ-trihydroxy-3.3′,5′-trimethoxy flavone (**2**), and 2,6,2′, 6′-tetramethoxy-4,4-bis(2,3-epoxy-1-hydroxypropyl)-biphenyl (**3**). We tested the antibacterial activity of pure compounds from strain 5-1-3 with the Oxford cup method. NRP echinomycin (**1**) showed excellent anti-MRSA activity with a minimum inhibitory concentration (MIC) of 2.0 μg/mL. Meanwhile, MIC of compound **2** and **3** was 128.0 μg/mL for both. In addition, 203 mg of echinomycin was isolated from 10 L of the crude extract broth of strain 5-1-3.

**Conclusion:**

In this study, *S. agglomeratus* 5-1-3 with strong resistance to MRSA was isolated from the soils in the Qinghai-Tibet Plateau. Strain 5-1-3 had a high yield of echinomycin (**1**) an NRP with a MIC of 2 μg/mL against MRSA. We propose that echinomycin derived from *S. agglomeratus* 5-1-3 may be a potent antibacterial agent for pharmaceutical use.

**Supplementary Information:**

The online version contains supplementary material available at 10.1186/s12934-023-02018-0.

## Background

Methicillin-resistant *Staphylococcus aureus* (MRSA) is one of the most common pathogenic bacteria in hospital and community infections [1, 2]. MRSA exhibits stronger infectivity than the Gram-positive cocci in any part of the human body, and the rate of infection incidence rises continuously [3]. A local MRSA infection takes a long time to treat, and once systemic infection occurs, the mortality rate is up to 20% [4, 5]. MRSA, except for methicillin resistance to other methicillin-beta-lactam classes, has the same strong resistance as ceftaroline [6], oxacillin [7], penicillin [8], and the cephalosporin class of antibiotics. Further, MRSA can change through various mechanisms involving aminoglycoside, large ring lactone class, tetracycline class, fluoroquinolone well ketone, sulfa, and rifampicin producing different levels of drug resistance [9, 10]. Vancomycin has a certain inhibitory effect on MRSA, and is commonly used in the clinical treatment of systemic infections caused by MRSA. However, with extensive application of vancomycin in clinical practice, vancomycin-resistant *Staphylococcus aureus* has been found in the United States [11]. Therefore, development of a new type of antibiotic with high efficiency and low MRSA toxicity, such as nonribosomal antibacterial peptides (NRAPs), is critical to the ability to limit infections.

Traditionally, NRAPs are characterized by chemical and mechanistic diversity, and they are an important source of discovery of novel antibiotics [12]. Most of the medically-important antibiotics are isolated from soil microbes [12]. Thanks to the rapid development of biotechnology, more of the previously-unrecognized and uncultured soil bacteria are being used as producers of NRAPs, such as lysocin E, teixobactin, and malacidins [12]. Thus, lysocin E was isolated from *Lysobacter* sp. RH2180-5 using the silkworm infection model. Meanwhile, teixobactin was characterized from uncultured *Eleheria terrae* [12]. This shows that uncultured bacteria are critical for the discovery of effective antibiotics. Uncultured bacteria account for approximately 99% of all species in the external environment [12–16], and among them, secondary metabolites of *Streptomyces* have been an important source of antibiotic discovery [17–19].

*Streptomyces* belongs to the phylum *Actinomycetes*. There are more than 1000 species of *Streptomyces* that are mainly found in soil. *Streptomyces* are filamentous Gram-positive bacteria reproducing mainly through spore formation; they are basically harmless to the human body. *Streptomyces* is an important antibiotic-producing genus, and approximately 2/3 of the antibiotics used in clinical practice are derived from *Streptomyces*, including streptomycin, tetracycline, erythromycin, neomycin, and kanamycin [17–19]. Research to culture more new *Streptomyces* is vital to the development of new antibiotics.

Some studies have shown that there may be undiscovered species of *Streptomyces* with antibacterial activity in soils in extreme environments that may provide new resources for the research and development of microbial natural products [20–25]. The Qinghai-Tibet Plateau in China is an example of an extreme environment, with most of the area located 3000–5000 m above sea level and an average elevation of more than 4000 m. The permafrost area of the Qinghai-Tibet Plateau is 147 × 10^4^ km^2^, accounting for more than 60% of the permafrost area in China. The average annual temperature in the hinterland of the plateau is below 0 ℃, and the warmest monthly average temperature is below 10 ℃ [26–28]. In this study, 36 *Streptomyces* strains with distinct colony morphological characteristics were isolated from 7 soil samples collected in the Qinghai-Tibet Plateau, and then 8 *Streptomyce*s strains resistant to MRSA were screened, and *S. agglomeratus* 5-1-3 was selected for further study based on results of the antibacterial activity test. Analysis of gene clusters of *S. agglomeratus* 5-1-3 showed that Clusters 3, 6, 12, 21, and 28 were used to synthesize NRPs. Therefore, the aim of this study was to search for NRPs with antibacterial activity among *S. agglomeratus* 5-1-3 secondary metabolites.

## Results

### Isolation and identification of *S. agglomeratus* 5–1-3

Strain 5-1-3 was isolated from soils in the Wuli region in the Qinghai-Tibet Plateau (N34°26′37.06ʹʹ, E92°43′41.37ʹʹ), China, at an elevation of 4595 m. Samples were obtained in April 2014. First, a square 5-point sampling method was used to collect soil samples to a depth of 0 to 30 cm, and then thoroughly mixed in a sterilized box. In accordance with the sampling principles for soil microbial analysis, all sampling processes and transport were kept sterile and samples were transported to the laboratory at − 20 ℃ after collection. Second, approximately 0.1 g of soil was dispersed in 0.9 mL of LB liquid medium and placed in 1.5 ml sterile centrifuge tubes. Samples were incubated at 30 °C and 100 r/min for 30 min and allowed to stand for 20 min. Then, using a tenfold gradient dilution to make a 10^− 3^ gradient, 200 µL of the suspension was spread on a Gauze No. 1 solid medium supplemented with nalidixic acid and cultivated at 28 °C under aerobic conditions. The isolated strain, showing a yellow basal mycelium, a white aerial mycelium, and a powdery surface on Gauze’s No. 1 solid medium, was purified and named strain 5-1-3. The pure culture was preserved in glycerol (25%) and stored at − 80 °C before use [29, 30]. As described previously [19], isolated strain 5-1-3 was identified with 16S rRNA-sequencing using primers F27 and R1492. The 16S rRNA sequence was compared with the NCBI database (https://www.ncbi.nlm.nih.gov/) to identify the most similar sequences, while phylogenetic trees were constructed using MEGA 5.0 based on the 16S rRNA sequence [31]. Evolutionary distance was calculated using the maximum composite likelihood method. The partial 16S rRNA gene sequence was submitted to the GenBank database and assigned accession number KF 729605.

### Growth and characterization of *S. agglomeratus* 5-1-3

**Fig. 1:**
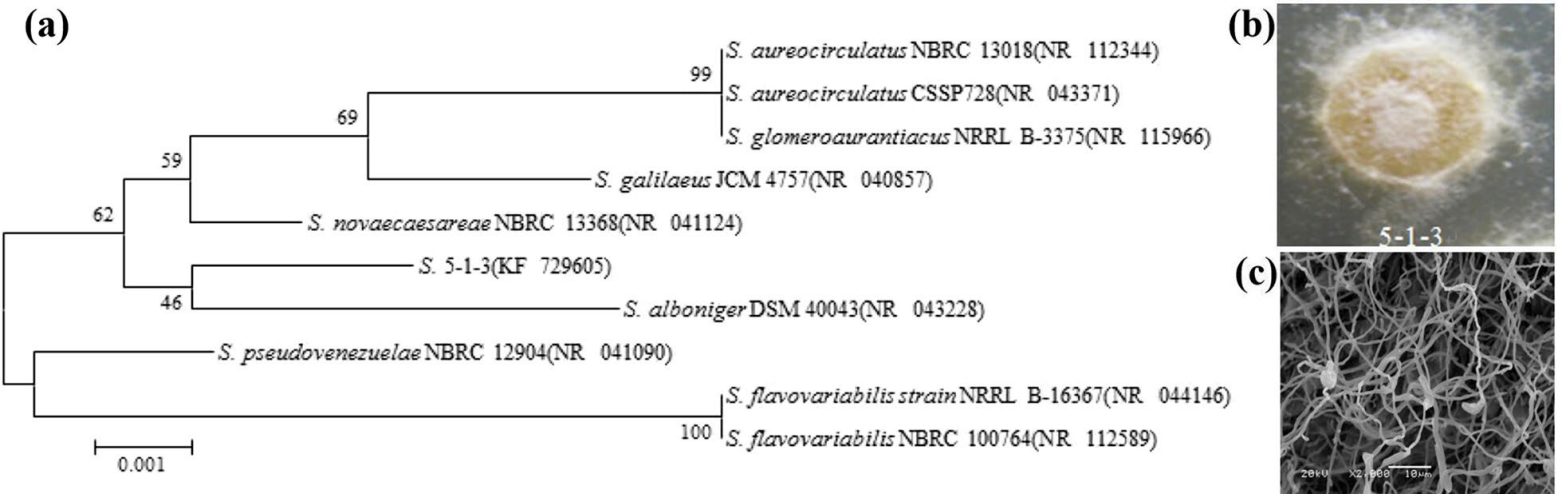
**a** Phylogenetic tree based on the 16S rRNA gene sequence, **b** characteristics of the colonies of *S. agglomeratus* 5-1-3, and **c** morphological diagram of *S. agglomeratus* 5-1-3

A yellow-white streptomycete designated as strain 5-1-3 was selected from 36 *Streptomyces* strains isolated from the Qinghai-Tibet Plateau, and an almost complete 16S rRNA gene sequence (1355 bp) was determined (Fig. 1a). The 16S rRNA gene sequence of strain 5-1-3 showed 99% similarity with *Streptomyces alboniger* DSM 40043. The best growth and yellow-white coloration of strain 5-1-3 were observed in Gauze’s No. 1 medium at 28 °C in subsequent experiments (Fig. 1b).

### Prediction analysis of secondary metabolite synthesis gene clusters in *S. agglomeratus* 5–1-3

**Fig. 2:**
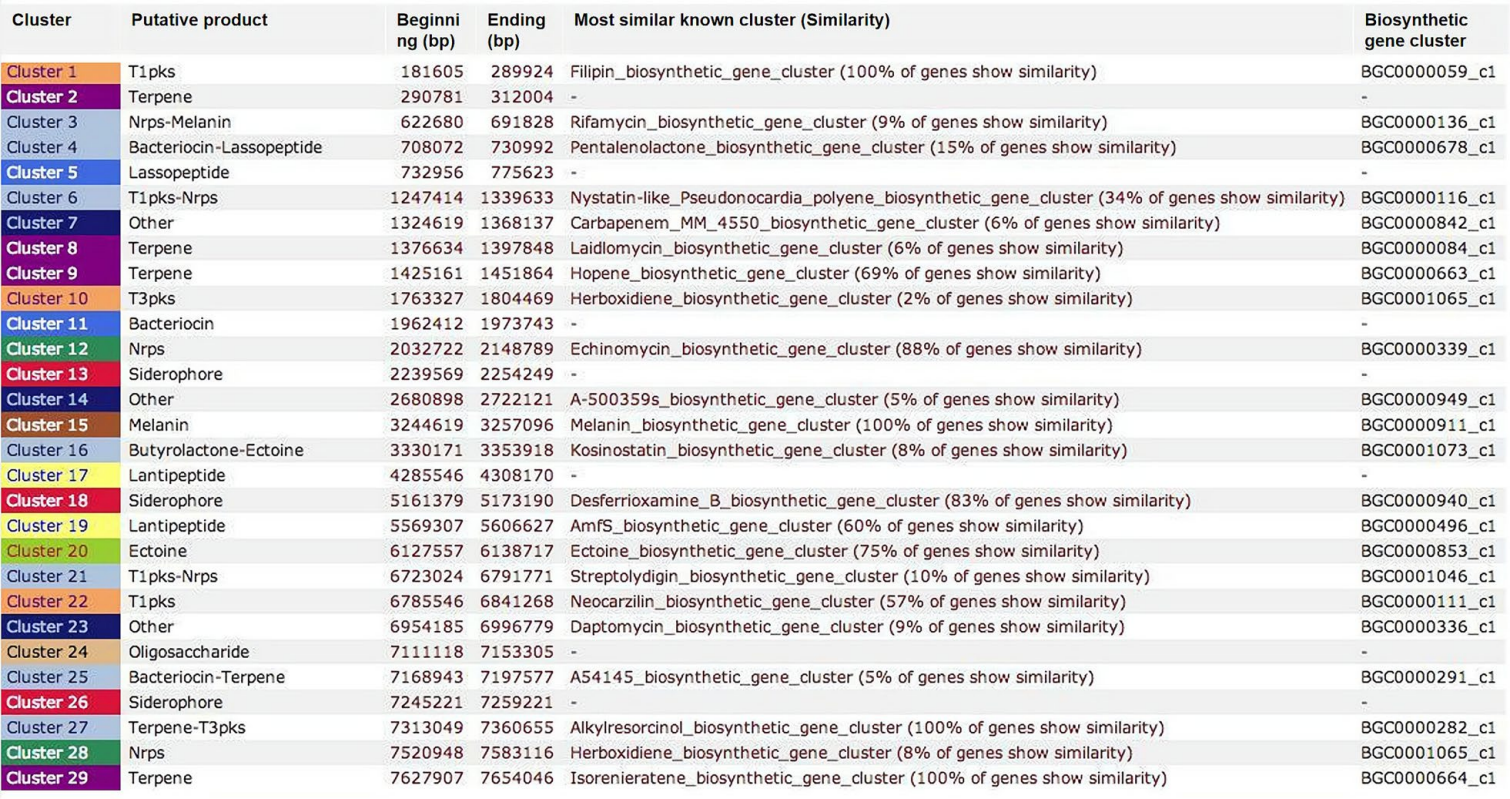
Metabolites predicted by antiSMASH in the *S. agglomeratus* 5-1-3 genome.

Antibiotic and secondary metabolite shell (antiSMASH) analysis showed that the whole genome of 5-1-3 contained 29 secondary metabolite synthesis gene clusters, and four of these gene clusters had 100% similarity, namely, Cluster 1, Cluster 15, Cluster 27, and Cluster 29 (Fig. 2). These four gene clusters were: filipin, synthesized by T1pks; melanin, synthesized by melanin; alkylresorcinol, synthesized by terpene-T3PKs; and isorenieratene, synthesized by terpene. In addition, seven gene clusters exhibited no predicted function or metabolites. Further analysis of gene clusters that synthesized NRPs showed that Clusters 3, 6, 12, 21, and 28 were used to synthesize rifamycin, nystatin-like, echinomycin, streptolydigin, and herboxidiene, respectively; Cluster 12 had an 88% likelihood to be used for the synthesis of echinomycin (Fig. 2), which was consistent with the results of the separation and identification of chemical constituents in this study.

### Antibacterial activity of *S. agglomeratus* 5-1-3

**Fig. 3:**
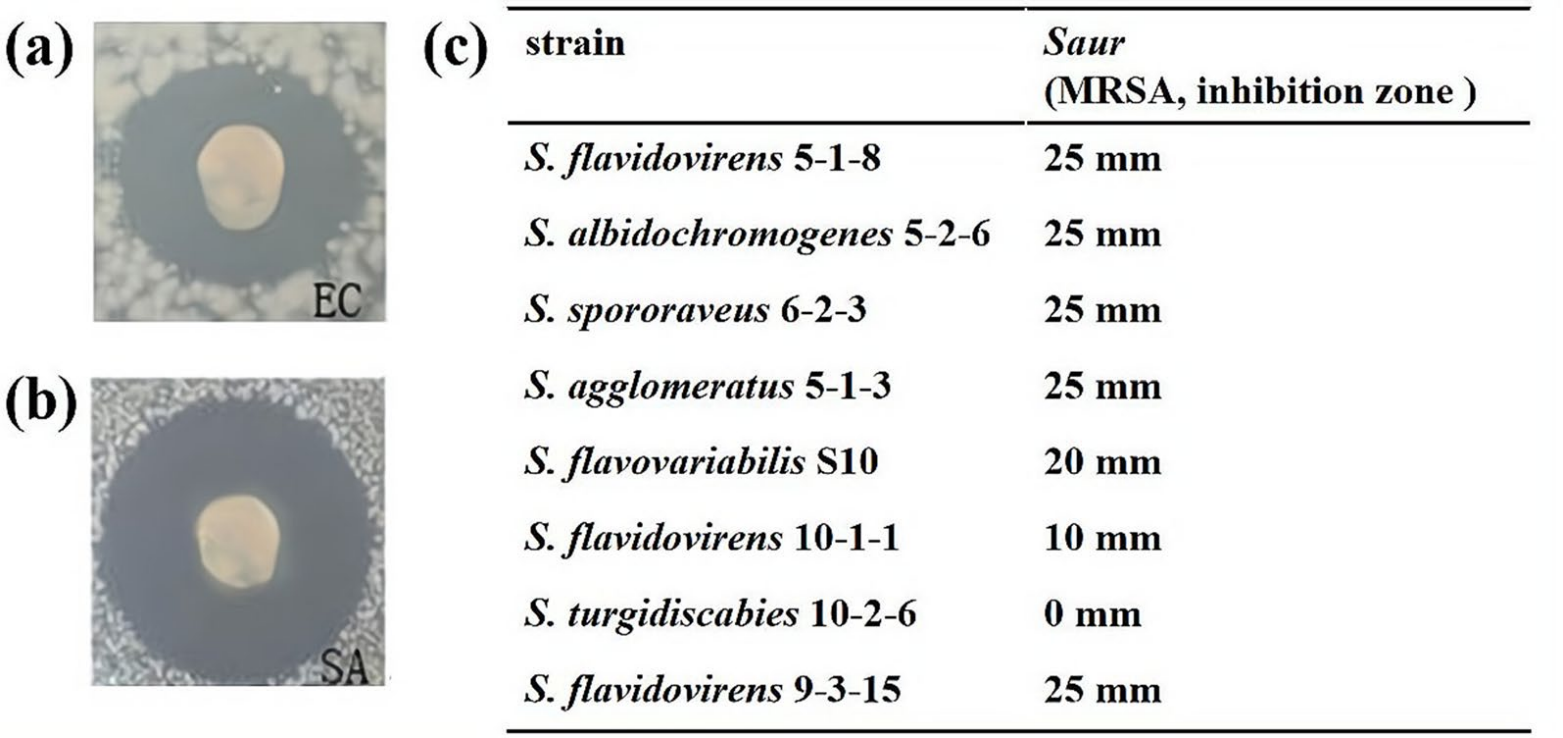
**a** Anti-*E. coli* activity of *S. agglomeratus* 5-1-3, **b** anti-*S. aureus* activity of *S. agglomeratus* 5-1-3, and **c** anti-MRSA activity of *S. agglomeratus* 5-1-3 and other *Streptomyces* isolates from soils of the Wuli region in Qinghai-Tibet Plateau

Antibacterial activity of extracts from strain 5-1-3 was tested with the Oxford cup method [32]. The indicator bacteria used in the antibacterial activity test were *Escherichia coli* (*E. coli*), *Staphylococcus aureus* (*S. aureus*), and MRSA. *E. coli* and *S. aureus* were obtained from the Key Laboratory of Extreme Environmental Microbial Resources and Engineering, while MRSA was obtained from the Centre for Molecular Biology, Swansea University School of Medicine, UK. Our results showed that the diameter of the inhibition zone of the extracts of strain 5-1-3 against the above pathogenic bacteria were 25 mm (Fig. 3).

### Purification, structural identification, and antibacterial activity of compounds from *S. agglomeratus* 5-1-3

Crude extract was extracted with ethyl acetate and purified with column chromatography and semipreparative HPLC. Compound **1** was isolated as a white, amorphous powder. The ^13^C NMR spectrum indicated 51 carbons which were attributed to eleven methyls, three methylenes, twenty-one methines, and sixteen quaternary carbons. These carbons included four N-CH_3_ (*δ*_C_ 29.8, 30.9, 31.5, 32.3) and two oxygenated methylenes (*δ*_C_ 64.7, 64.9); therefore, we concluded that there were 2 serine units in this cyclic peptide. There were also six aromatic ring quaternary carbon signals (*δ*_C_ 144.1, 144.1, 142.3, 142.4, 143.6, 143.6), ten aromatic tertiary carbons (*δ*_C_ 129.3, 129.4, 129.7, 129.7, 130.9, 131.0, 131.9, 132.0, 140.0, 140.1), and two carbonyl carbons (*δ*_C_ 167.3, 167.6); based on literature, we identified them as two quinoxaline-2 carboxylic acid residues. A comprehensive analysis of ^1^H and ^13^C NMR data of the compound showed that cyclopeptide compound **1** contained 2 alanines, 2 N-methylvaline, 2 serines, 2 sulfur-containing amino acids, and 2 quinoxalin-2 carboxylic acids. Based on NMR spectroscopic analyses (Table 1, Additional file 1: Figs. S1-S2) and by comparisons with data reported in the literature, compound **1** was identified as echinomycin [33], it was an NRP.

**Table 1:** ^13^C NMR Spectroscopic Data of Compounds **1 ~ 3** [100 MHz,δ(ppm)]

Position	**1 (***δ*_C_, CD_3_Cl**)**	Position	**2 (***δ*_C_, DMSO**)**	Position	**3 (***δ*_C_, CD_3_Cl**)**
1,1′	129.3, 129.4	2	138.2	1,1′	132.1
2,2′	131.9, 132.0	3	122.7	2,2′	147.2
3,3′	129.7, 129.7	4	178.3	3,3′	102.7
4,4′	130.9, 131.0	5	104.3	4,4′	134.3
5,5′	144.1, 144.1	6	161.7	5,5′	102.7
6,6′	142.3, 142.4	7	99.0	6,6′	147.2
7,7′	140.0, 140.1	8	164.6	7,7′	85.9
8,8′	143.6, 143.6	9	94.3	8,8′	54.3
9,9′	167.3, 167.6	10	156.8	9,9′	71.8
10,10′	53.4, 53.5	11	121.2	4 × OMe	56.4
11,11′	64.7, 64.9	12	112.5		
12,12′	168.8, 168.8	13	155.9		
13,13′	61.9, 62.7	14	116.1		
14,14′	20.4, 20.4	15	150.2		
15,15′	17.1, 17.1	16	112.5		
16,16′	18.1, 18.1	OMe	60.2		
17,17′	31.5, 32.3	OMe	60.2		
18,18′	170.9, 171.1	OMe	56.2		
19,19′	60.0, 61.9				
20,20′	29.8, 30.9				
21,21′	173.4, 173.5				
22,22′	51.9, 52.3				
23,23′	18.8, 19.0				
24,24′	170.2, 170.2				
25,25′	46.6, 27.8				
26	15.2				

Compound **2** was isolated as a yellow, amorphous powder. The ^13^C NMR spectrum (Additional file 1: Fig. S4) indicated 18 carbons which were attributed to nine aromatic ring quaternary carbon signals (*δ*_C_ 104.3, 116.1, 121.2, 138.2, 150.2, 155.9, 156.8, 161.7, 164.6), four aromatic tertiary carbons (*δ*_C_ 94.3, 99.0, 112.5, 112.5), one carbonyl carbon (*δ*_C_ 178.3), and three methoxyl carbons (*δ*_C_ 56.2, 60.2, 60.2). Using this information, the analysis of the ^1^H-NMR spectrum (Additional file 1: Fig. S3), and comparisons with data reported in the literature, we established the structure of compound **2** as 5,7,4′-trihydroxy-3.3′,5′-trimethoxyflavone [34].

**Fig. 4:**
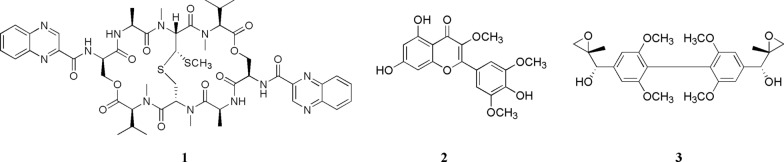
Structures of compounds **1–3**: echinomycin (**1**), flavones (**2**), and biphenylneolignan (**3**)

Compound **3** was isolated as a yellow oil. The ^13^C NMR spectrum (Additional file 1: Fig. S6) indicated 12 carbons which were attributed to four aromatic ring quaternary carbon signals, two aromatic tertiary carbons, one methyl, one oxygenated methylene, one oxygenated methine, one oxygenated quaternary carbon, and two methoxyl carbons. The ^1^H NMR spectrum (Additional file 1: Fig. S5) of **3** indicated presence of two substituted benzene rings which were symmetrical, two methoxyl groups, and an allylic alcohol. Presence of a secondary and a tertiary carbon signal at *δ*_C_ 71.8 and 54.3, respectively, in the ^13^C NMR spectrum of **3** indicated presence of an epoxide as a terminal moiety. Using this information, we established the structure of compound **3** as 2,6,2′,6′-tetramethoxy-4,4-bis(2,3-epoxy-1-hydroxypropyl)-biphenyl [35] (Fig. 4).

Vancomycin has a certain inhibitory effect on MRSA and it is commonly used in clinical treatment of systemic infections caused by MRSA. However, with the extensive application of vancomycin in clinical practice, vancomycin-resistant *Staphylococcus aureus* has been found in the United States [11].

**Fig. 5:**
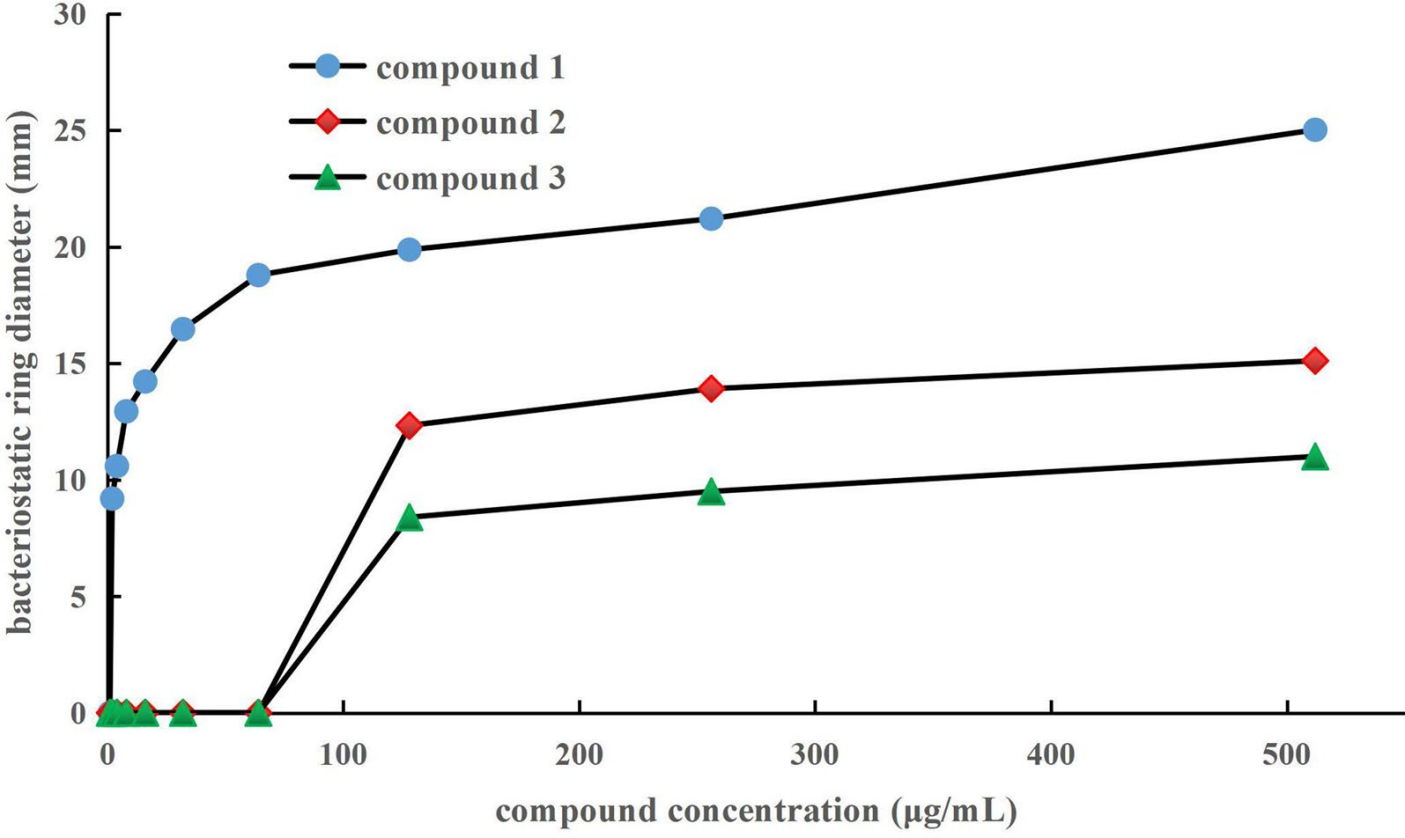
Anti-MRSA activity of compounds **1**–**3**

Antibacterial activity of compounds **1–3** was tested with the Oxford cup method. Echinomycin (**1**) exhibited excellent anti-MRSA activity with a MIC of 2.0 μg/mL (Table 2, Fig. 5). This is a very valuable result of antibacterial activity, and it indicates that echinomycin can be used as a lead antibacterial compound, with significant medical potential. Meanwhile, MIC of compounds **2** and **3** was 128.0 μg/mL for both, indicating that the activity of NRPs was better than in other compounds.

**Table 2:** Bacteriostatic ring diameter of compounds **1** ~ **3** for MRSA

compound concentration (μg/mL)	bacteriostatic ring diameter (mm)		
	compound **1**	compound **2**	compound **3**
1.0	-	-	-
2.0	9.20 ± 0.59	-	-
4.0	10.60 ± 0.32	-	-
8.0	12.93 ± 0.82	-	-
16.0	14.21 ± 0.51	-	-
32.0	16.46 ± 1.09	-	-
64.0	18.78 ± 0.92	-	-
128.0	19.87 ± 0.88	12.32 ± 0.46	8.40 ± 0.51
256.0	21.20 ± 0.41	13.90 ± 0.54	9.51 ± 0.51
512.0	25.02 ± 1.59	15.10 ± 0.50	11.01 ± 1.19

“-” means no bacteriostatic ring diameter, data of bacteriostatic ring diameter were “mean ± standard deviation” of 3 replicates; if the diameter of antibacterial ring was > 8.0 mm, it was taken to have antibacterial activity. Sterile water was the control, and no bacteriostatic ring was detected.

## Discussion

Since the first report of MRSA by Jevons in 1961, MRSA has become a common pathogenic bacterium in clinical practice, especially in postoperative infections. It is also a common superbacterium, greatly challenging clinical treatment [1]. MRSA bacteria expressed a variety of antibiotic resistance-related genes and exhibited different degrees of resistance to *β*-lactam, quinolone, aminoglycoside, tetracycline, and macrolide antibiotics. In addition, with the extensive application of vancomycin in clinical practice, vancomycin-resistant *Staphylococcus aureus* has been found in the United States [11]. Therefore, development of a new type of antibiotic with high efficiency and low MRSA toxicity is critical to the ability to limit infections.

Natural compounds exhibit a diversity of chemical structures and biological activities, and are an important source of discovery of new high-efficiency and low-toxicity antibiotics. Approximately 70% of the nearly 10,000 kinds of natural antibiotics discovered thus far are produced by actinomycetes, and antibiotics derived from *Streptomyces* accounted for 52% of the total [36–39]. However, recent studies have shown that only new strains of *Streptomyces* lead to discoveries of new antibiotics [16, 40–42].

Some actinomycete groups in extreme environments may produce unique primary and secondary metabolites providing new resources for the research and development of microbial natural products. For example, Taddei et al. isolated 71 strains of Streptomyces from different soil samples in Venezuela, of which 67 strains were new species [23]. Kim et al. also isolated two new Streptomyces species from dry soils in Northumberland, UK [24].

Permafrost in the Qinghai-Tibet Plateau is characterized by long periods of low temperatures and magnetic radiation, and a severe lack of liquid water and available nutrients for biological survival. It constitutes, therefore, an important example of extreme environments. Due to a low level of human interference, it has become a significant source of extreme-environment microbes. Zhang found that 41 *Streptomyces* strains collected from the Qinghai-Tibet Plateau were significantly antagonistic to *E. coli*, *S. aureus,* and *Bacillus subtili*s, indicating that there were strong antagonistic *Streptomyces* strains in the soil of the Qinghai-Tibet Plateau [43]. Ma found that the abundant physiological diversity and secondary metabolites of Streptomycetes isolated from the permafrost of the Tibetan Plateau may have potential implications for biotechnology and biological products [44].

NRPs refer to peptide compounds that assemble natural or nonnatural amino acids or modify amino acids through modular nonribosomal peptide synthetases [13]. Efficient and flexible NRPSs ensure structural diversity of synthetic NRAPs [14]. Meanwhile, modification of NRAPs not only includes cyclization or introduction of heterozygous cyclized molecules, but also glycosylation, acylation, lipidylation, and other pathways [15].

NRAPs exhibit unique antibacterial mechanisms. For example, the combination of daptomycin and Ca^2+^ in a 1:1 ratio can be transported to the surface of the cell membrane, and be further dispersed where they form ion channels on the surface of bacteria leading to ion outflow. In addition, NRAPs are not susceptible to resistance, as shown with some of the new NRAP target molecules under development. For example, teixobactin is a condensation peptide composed of methylphenylalanine and a four-amino acid isomeric phenolic ester, it kills pathogens that cause wounds and invasive infections, such as *Staphylococcus aureus*, MRSA, and pneumonia caused by *Streptococcus* and *Mycobac terium tuberculosis*, and it also exhibit reasonable antibacterial activity against the difficult clostridium and carbon jaundice bacillus [16]. Lugdunin, a macrocyclic thiazolyl, has extensive and effective antibacterial activity with MIC values ranging from 1.5–12 ug/mL against Gram-positive bacteria, including opportunistic pathogens, such as MRSA and vancomycin-resistant Enterococcus which are difficult to treat with conventional antibiotics [12]. In addition, Ilamycins composed of rare L-3-nitrotyrosine and L-2-amino-4-hexenoic acid showed potent antituberculous activity with MIC of 9.8 nM [12]. Therefore, NRPs have attracted much attention in the fight against superbacteria, and are now an important source of new antibiotic research and development.

In this study, antiSMASH analysis showed that the whole genome of strain 5-1-3 contained 29 secondary metabolite synthesis gene clusters. Further analysis of the gene clusters that synthesize NRP substances showed that Clusters 3, 6, 12, 21, and 28 were used to synthesize rifamycin, nystatin-like, echinomycin, streptolydigin, and herboxidiene, respectively, and Cluster 12 had an 88% likelihood of being used to synthesize echinomycin. Therefore, it is possible to search for NRPs from strain 5-1-3, as shown with the results of chemical composition isolation and identification in this study.

MRSA showed some resistance to erythromycin, rifampicin, and vancomycin. Therefore, antibiotics commonly used in hospitals have poor bactericidal efficacy against MRSA. The antibacterial activity of compounds **1**–**3** was tested with the Oxford cup method. Echinomycin (**1**) showed excellent anti-MRSA activity with an MIC of 2.0 μg/mL. This is a very valuable antibacterial activity result, which indicates that echinomycin can be used as a lead antibacterial compound in which to study the active group to provide sources for the development of new active molecules against MRSA.

## Conclusions

We characterized *S. agglomeratus* 5-1-3 isolated from the soils of the Wuli region in the Qinghai-Tibet Plateau and evaluated anti-MRSA activity of its secondary metabolites. AntiSMASH analysis showed that the whole genome of strain 5-1-3 contained 29 secondary metabolite synthesis gene clusters, of which 5 were related to the synthesis of NRPs. A further chemical investigation of the extract of strain 5-1-3 led to the discovery of one NRP, elucidated as echinomycin, a good antibacterial agent, and two other types of compounds, 5,7,4′-trihydroxy-3.3′,5′-trimethoxy.

flavone and 2,6,2′,6′-tetramethoxy-4,4′-bis(2,3-epoxy-1-hydroxypropyl)-biphenyl, both displaying antibacterial activities. We conclude that strain 5-1-3 can be used as a new source of NRPs, and compound **1** can be used as a starter molecule against MRSA for further studies on the structure–activity relationships.

## Methods

### Prediction of the secondary metabolite synthesis gene cluster of *S. agglomeratus* 5-1-3

The antibiotics and secondary metabolites corresponding to the potential secondary metabolite synthesis gene cluster in strain 5-1-3 were analyzed through the online website (http://antismash.secondarymetabolites.org/, antiSMASH). First, we selected “Submit Bacterial sequence” option, second, we entered the NCBI accession number corresponding to strain 5-1-3 or the full genome sequence in FASTA format. Three parameters were selected for analysis: ClusterFinder, the ClusterFinder Algorithm for BGC Border Prediction, and Extra Features. The Extra Features analysis includes KnownClusterBlast, SubClusterBlast, and ActiveSiteFinder.

### Assessment of antibacterial activity

We tested the antibacterial activity of extracts and pure compounds from strain 5-1-3 with the Oxford cup method. The indicator bacteria used in the antibacterial activity test were *E. coli*, *S. aureus*, and MRSA. *E. coli* and *S. aureus* were obtained from the Key Laboratory of Extreme Environmental Microbial Resources and Engineering, and MRSA was obtained from the Centre for Molecular Biology, Swansea University School of Medicine, UK.

The test method was as follows. First, bacterial colonies were selected from the slant culture medium of the test bacteria and inoculated into 50 mL sterile normal saline solution. After 24 h at 37 °C, the bacterial solution was diluted to an OD_600_ value of approximately 0.4 for later use. The extract or pure compound of each experimental group was dissolved in methanol to prepare a 5 mg/mL test solution. 15 mL of the sterilized solid medium of hot LB agar was poured into petri dishes (lower layer), and allowed to solidify. In addition, another sterilized solid medium of hot LB agar was cooled to approximately 50 °C and mixed with test bacteria, and 5 mL of the medium mixed with bacteria were added to the solidified medium (upper layer). The Oxford cup (inner diameter of 6 mm, outer diameter of 8 mm) was aseptically placed vertically on the surface of the medium and gently pressurized to ensure a seal with the medium, and 100 μL of extract or pure compound test solution was added into the cup without overflow. Then, the culture was placed at 37 °C for 24 h. The diameter of the inhibition zone was measured with Vernier calipers. All tests were repeated three times. Data were processed in SPSS (PASW Statistics 18), and the results were expressed as means ± standard deviation (SD).

### Purification and structural identification of the bioactive compound

Bacterial colonies were grown in Gauze’s No. 1 solid medium in a 1 L conical flask and maintained in a shaking incubator (120 rpm and 28 °C) for 30 days. Bacterial cells were collected following centrifugation at 1200 rpm for 10 min. We used 100% (v/v) ethyl acetate to extract the crude extract from collected 5-1-2 strain until the strain became colorless. The crude extract was separated and purified on a silica gel column (200–300 mesh) and eluted with gradient mixtures of chloroform–ethyl acetate (5:1, 2:1, 1:1, 1:3 v/v) to give four fractions (A–D). We then performed repeated separation of fraction A with Sephadex LH-20 (CHCl_3_: MeOH, 1:1). Fraction A-1 was purified using semipreparative high-performance liquid chromatography (HPLC) with a C18 column (SPODS-A, 20 × 250 mm, 5 μm, Hanbon, China) using a 6:4 (2.0 mL/min) ratio of acetonitrile to distilled water to obtain compound **1**. Fraction C was purified using semipreparative HPLC (H_2_O − MeOH, 1:3, 2.0 mL/min) to obtain compounds **2** and **3**.

The pure active compound was characterized with NMR analyses and comparisons with the literature. Structural identification of the purified compound was clarified with a DRX-400 spectrometer (Bruker, Rheinstetten, Germany) using spectroscopic techniques for ^1^H and ^13^C. The units of the chemical shifts are ppm(*δ*), and residual CHCl_3_ (*δ*_H_ 7.26, *δ*_C_ 77.0), and DMSO (*δ*_H_ 2.50, *δ*_C_ 39.5) were used as internal standards.

## Supplementary Information

**Additional file 1**: **Figure S1** ^1^H NMR spectrum of compound **1** in CDCl_3_ (400 MHz). **Figure S2** ^13^C NMR spectrum of compound **1** in CDCl_3_ (100 MHz). **Figure S3** ^1^H NMR spectrum of compound **2** in DMSO (400 MHz). **Figure S4** ^13^C NMR spectrum of compound **2** in DMSO (100 MHz). **Figure S5** ^1^H NMR spectrum of compound **3** in CDCl_3_ (400 MHz). **Figure S6** ^13^C NMR spectrum of compound **3** in CDCl_3_ (100 MHz).

## Data Availability

All data generated or analyzed during the study are included in this paper and Additional file 1.

## Abbreviations

NRPs: Nonribosomal peptides
NRAPs: Nonribosomal antibacterial peptides
MRSA: Methicillin-resistant *Staphylococcus aureus*
MIC: Minimum inhibitory concentration
HPLC: High-performance liquid chromatography
antiSMASH: Antibiotics and secondary metabolite analysis shell
