# Supplementary material for: Nonribosomal antibacterial peptides isolated from Streptomyces agglomeratus 5-1-3 in the Qinghai-Tibet Plateau
Source: Microb Cell Fact. 2023 Jan 6;22:5. doi: 10.1186/s12934-023-02018-0 (PMC9824969; doi:10.1186/s12934-023-02018-0)
Supplement: Supplementary file 1 — Additional file 1: Figure S1 1H NMR spectrum of compound 1 in CDCl3 (400 MHz). Figure S2 13C NMR spectrum of compound 1 in CDCl3 (100 MHz). Figure S3 1H NMR spectrum of compound 2 in DMSO (400 MHz). Figure S4 13C NMR spectrum of compound 2 in DMSO (100 MHz). Figure S5 1H NMR spectrum of compound 3 in CDCl3 (400 MHz). Figure S6 13C NMR spectrum of compound 3 in CDCl3 (100 MHz). [file 12934_2023_2018_MOESM1_ESM.doc]

**Nonribosomal antibacterial peptides isolated from *Streptomyces agglomeratus* 5-1-3 in the Qinghai-Tibet Plateau**

Kan Jiang^1*^, Ximing Chen^2,3^, Wei Zhang^2,3^, Yehong Guo^1^, Guangxiu Liu^2,3*^

*Corresponding author: Tel: 18919199825; 13609379548

1. *mail address*: [jiangk19@126.com](mailto:jiangk19@126.com); liugx@lzb.ac.cn


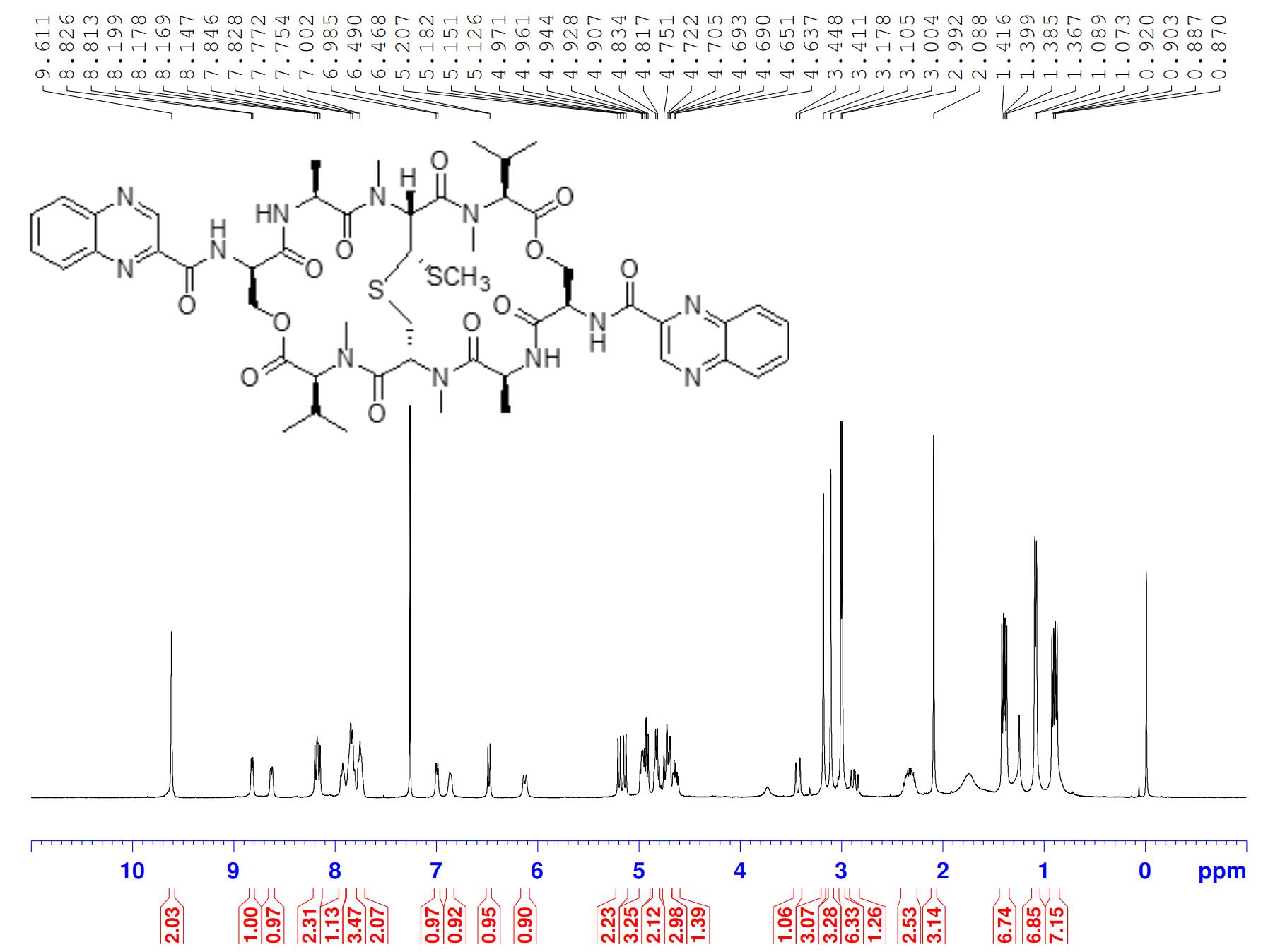


Fig. S1 ^1^H NMR spectrum of compound **1** in CDCl_3_ (400 MHz)


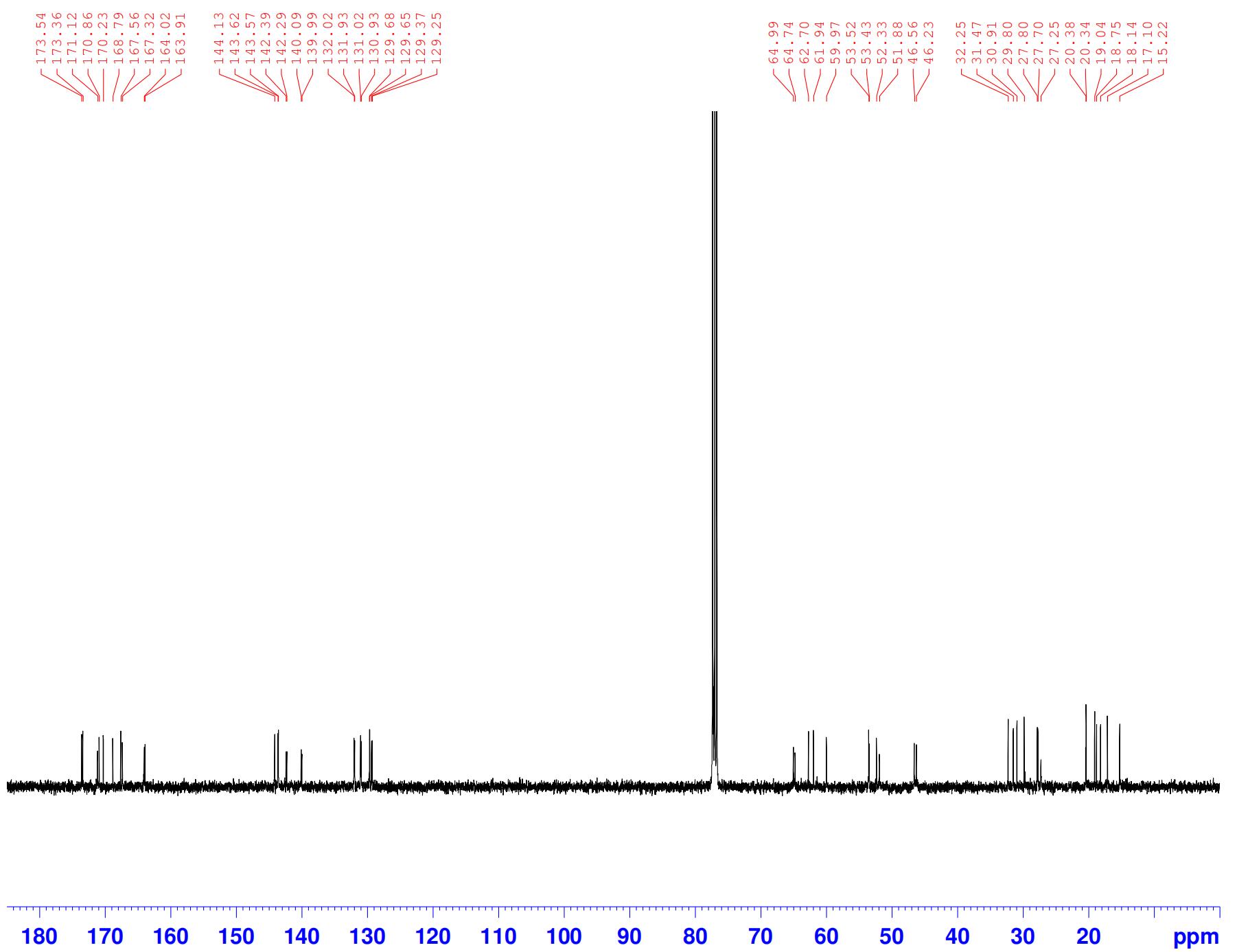


Fig. S2 ^13^C NMR spectrum of compound **1** in CDCl_3_ (100 MHz)


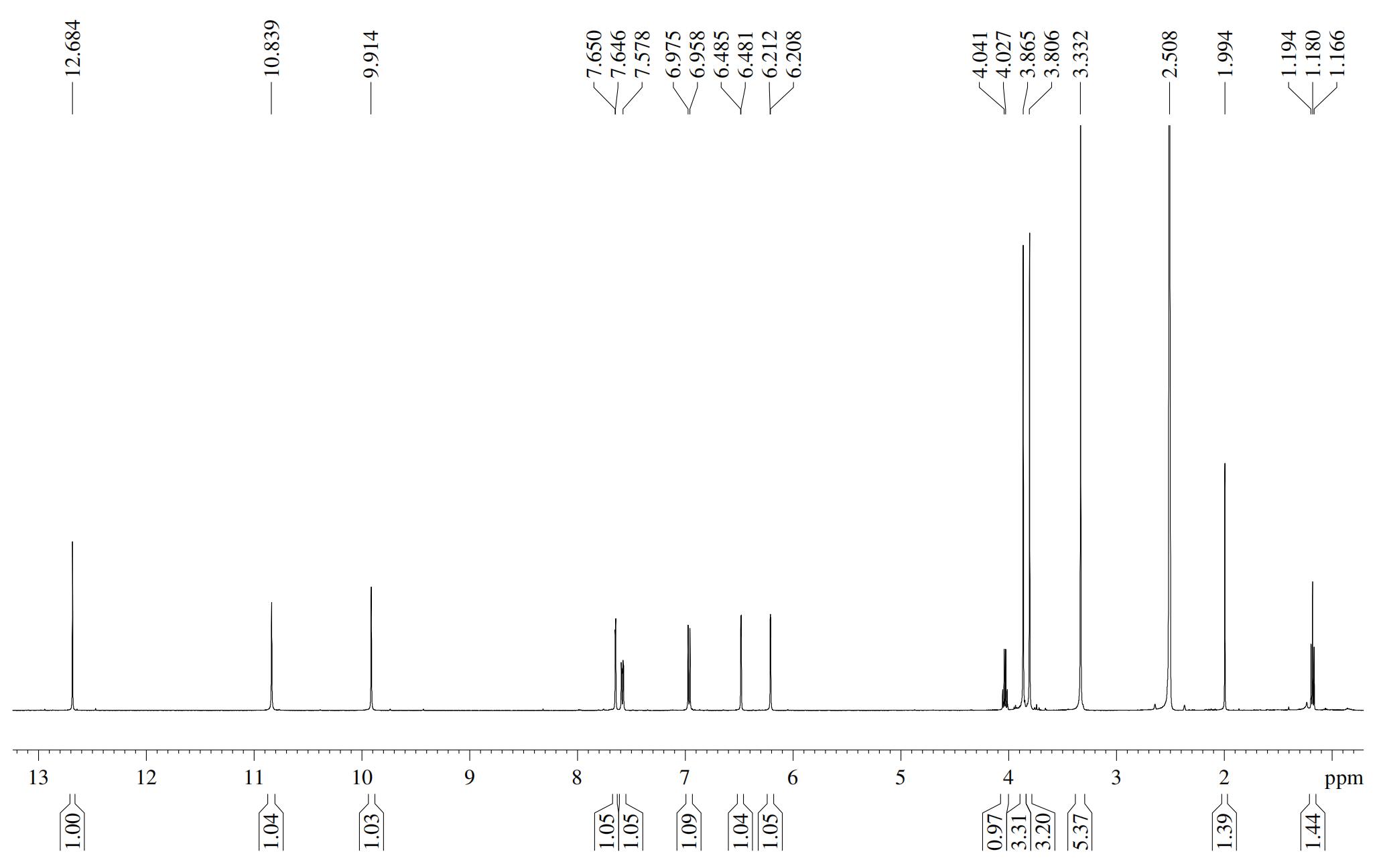


Fig. S3 ^1^H NMR spectrum of compound **2** in DMSO (400 MHz)


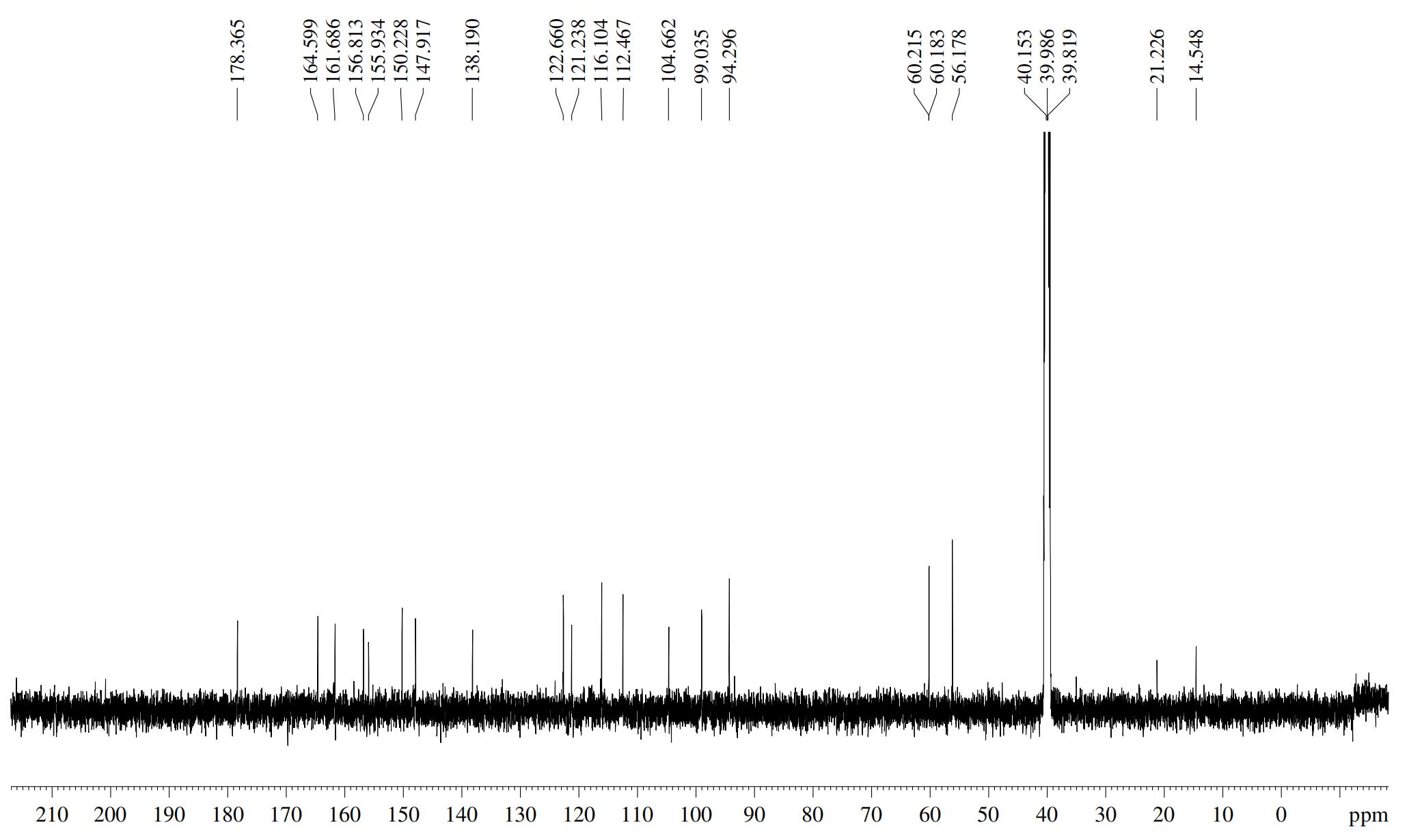


Fig. S4 ^13^C NMR spectrum of compound **2** in DMSO (100 MHz)


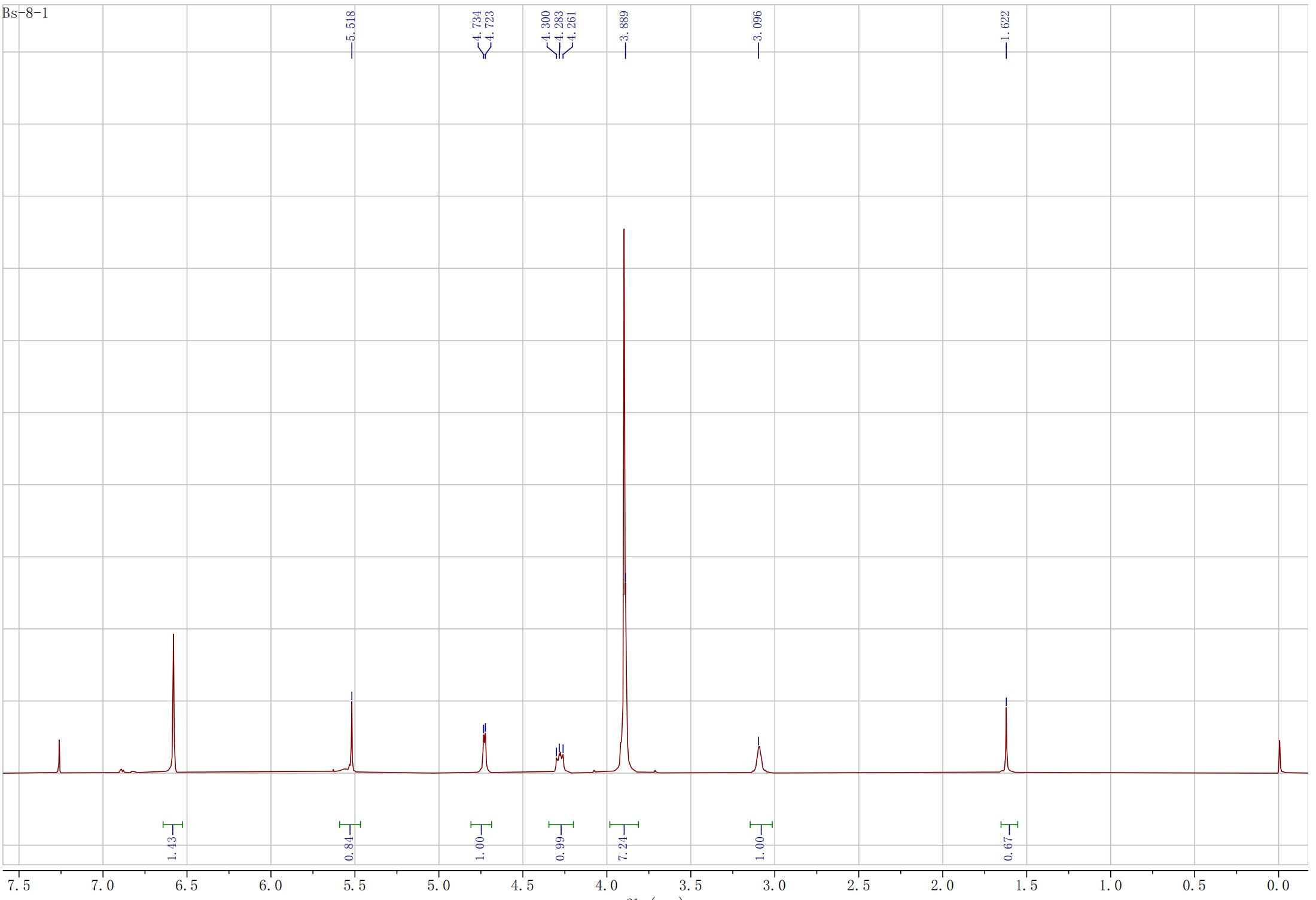


Fig. S5 ^1^H NMR spectrum of compound **3** in CDCl_3_ (400 MHz)


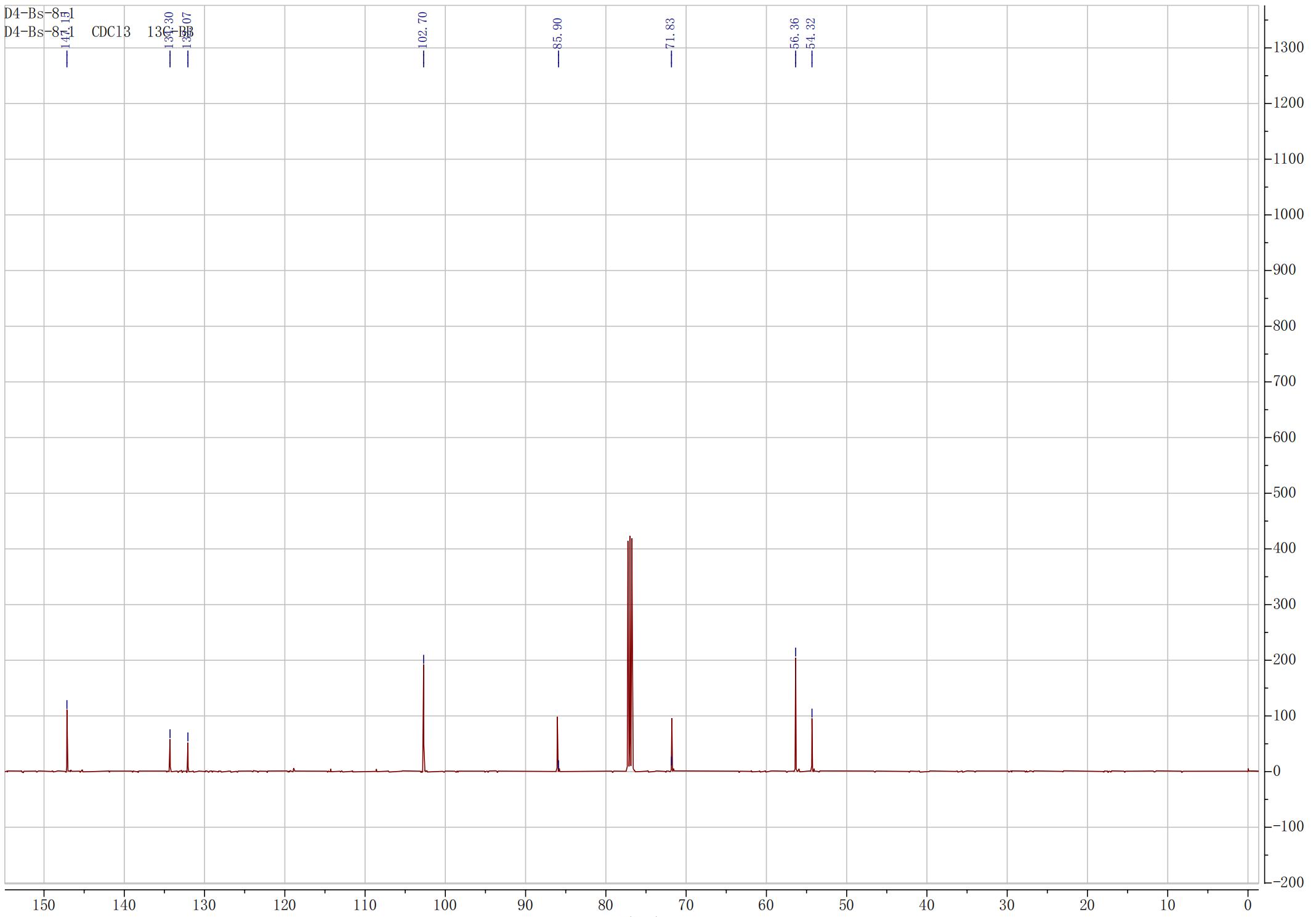


Fig. S6 ^13^C NMR spectrum of compound **3** in CDCl_3_ (100 MHz)
